# Vinegar/Tetramethylpyrazine Induces Nutritional Preconditioning Protecting the Myocardium Mediated by VDAC1

**DOI:** 10.1155/2021/6670088

**Published:** 2021-04-20

**Authors:** Huan He, Liang Wang, Yang Qiao, Qing Zhou, Bin Yang, Lu Yin, Dong Yin, Ming He

**Affiliations:** ^1^Institute of Cardiovascular Diseases, Jiangxi Academy of Clinical Medical Sciences, The First Affiliated Hospital of Nanchang University, Nanchang 330006, China; ^2^Jiangxi Provincial Key Laboratory of Basic Pharmacology, Nanchang University School of Pharmaceutical Science, Nanchang 330006, China; ^3^Department of Rehabilitation, The First Affiliated Hospital of Nanchang University, Nanchang 330006, China; ^4^Bioprocessing Technology Institute, Agency for Science, Technology and Research, Singapore, Singapore 138668; ^5^Jiangxi Provincial Key Laboratory of Molecular Medicine, The Second Affiliated Hospital, Nanchang University, Nanchang 330006, China

## Abstract

Vinegar is good for health. Tetramethylpyrazine (TMP) is the main component of its flavor, quality, and function. We hypothesized that vinegar/TMP pretreatment could induce myocardial protection of “nutritional preconditioning (NPC)” by low-dose, long-term supplementation and alleviate the myocardial injury caused by anoxia/reoxygenation (A/R). To test this hypothesis, TMP content in vinegar was detected by HPLC; A/R injury model was prepared by an isolated mouse heart and rat cardiomyocyte to evaluate the myocardial protection and mechanism of vinegar/TMP pretreatment by many enzymatic or functional, or cellular and molecular biological indexes. Our results showed that vinegar contained TMP, and its content was in direct proportion to storage time. Vinegar/TMP pretreatment could improve hemodynamic parameters, decrease lactate dehydrogenase (LDH) and creatine phosphokinase activities, and reduce infarct size and apoptosis in the isolated hearts of mice with A/R injury. Similarly, vinegar/TMP pretreatment could increase cell viability, decrease LDH activity, and decrease apoptosis against A/R injury of cardiomyocytes. Vinegar/TMP pretreatment could also maintain the mitochondrial function of A/R-injured cardiomyocytes, including improving oxygen consumption rate and extracellular acidification rate, reducing reactive oxygen species generation, mitochondrial membrane potential loss, mitochondrial permeability transition pore openness, and cytochrome c releasing. However, the protective effects of vinegar/TMP pretreatment were accompanied by the downregulation of VDAC1 expression in the myocardium and reversed by pAD/VDAC1, an adenovirus that upregulates VDAC1 expression. In conclusion, this study is the first to demonstrate that vinegar/TMP pretreatment could induce myocardial protection of NPC due to downregulating VDAC1 expression, inhibiting oxidative stress, and preventing mitochondrial dysfunction; that is, VDAC1 is their target, and the mitochondria are their target organelles. TMP is one of the most important myocardial protective substances in vinegar.

## 1. Introduction

**Figure 1:**
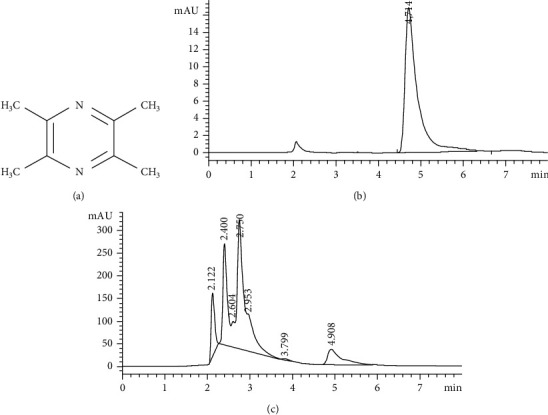
TMP concentration test in 6 kinds of commercial food vinegar. (a) Chemical structures of TMP. (b) Standard solution of TMP. (c) A typical vinegar sample of TMP.

Vinegar, produced via the complex fermentation of grain or fruits, is a worldwide popular condiment [1, 2]. Many works have shown the beneficial effects of vinegar consumption on the health of human [3–7]. Tetramethylpyrazine (TMP, Figure 1(a)), which naturally exists in a variety of fried, roasted, or fermented foods, is considered to be the main component of vinegar's flavor, nutrition, and health care function and quality [1, 2, 8]. TMP is also called as ligustrazine, derived from the rhizome of *Ligusticum wallichii*, and has many biological activities [9]. More and more medical scientists believe that TMP is beneficial to human health, especially cardiovascular and cerebrovascular health [10–12]. Previously, we have found that TMP has an excellent protection on a variety of the myocardium or blood vessel injuries [13–16].

Coronary artery spasm or occlusion may result in myocardial ischemia, or even infarction [17]. In clinical practices, fast coronary flow reconstruction and reperfusion is the first-line treatment. However, reperfusion may lead to more serious tissue injury than the ischemia itself (ischemia/reperfusion, I/R) [18]. Ischemic preconditioning (IPC) and pharmacological preconditioning (PPC) could alleviate myocardial damage [17–19], but the application of these methods is limited by ethical concerns and technical difficulties. We have previously raised the concept of nutritional preconditioning (NPC) and proved it to be as an ideal solution to relieve myocardial damage [20–24]. We have further revealed that NPC inhibits intracellular reactive oxygen species (ROS) generation and mitochondria-mediated apoptosis pathway, which might efficiently alleviate anoxia/reoxygenation (A/R) damage, prevent mitochondrial dysfunction, and improve cardiac function [18, 22–24].

Voltage-dependent anion channel 1 (VDAC1) is a protein on the outer membrane of the mitochondria. VDAC1 is involved in the construction of mitochondrial permeability transition pore (mPTP) and acts as the gatekeeper of mPTP [25]. In responses to A/R, VDAC1 expression is upregulated, mPTP is opened, and myocardial damage is aggravated. We have shown that VDAC1 plays an important role in the resveratrol's protection against A/R damage; in other words, VDAC1 was the target of resveratrol myocardial protection [20, 26, 27].

Therefore, we intend to explore whether NPC through low-dose, long-term supplementation of vinegar/TMP, representing a healthy dietary habit, could induce myocardial protection, and whether the protection mechanism is mediated by VDAC1 on the mitochondria.

## 2. Materials and Methods

### 2.1. Reagents and Vinegar Samples

Vinegars A-F were purchased from supermarket. TMP (purity: 98%) and atractyloside (Atr) were purchased from Sigma-Aldrich (St. Louis, MO, USA). pAD/VDAC1 was purchased from Gene Chem Co., Ltd. (Shanghai, China). Antibodies against VDAC1, cytochrome c (cyt C), and *β*-actin were purchased from Cell Signaling Technology (Beverly, MA, USA).

### 2.2. Animals

Adult male Kunming mice (20-22 g) and the neonatal (0-3 days) Sprague-Dawley (SD) rats were purchased through the Animal Center of Nanchang University (Nanchang, China). The animal protocols complied with the NIH *Guide for the Care and Use of Laboratory Animals* (NIH Publication No. 85-23, revised 1996) and were approved by the ethics committee of Nanchang University (No. 2019-0106).

### 2.3. Determination of TMP in Vinegar Samples

TMP control stock solution, negative control solution, and test solution were weighed and filtered through the 0.22 *μ*m organic membrane. The TMP content in the vinegar samples was measured with HPLC as described previously [28]. A HPLC system (Agilent 1100 HPLC Systems, Santa Clara, CA, USA) with a Chemstation Edition Workstation, aG1313A autosampler, and Hypersil ODS (Thermo, Waltham, MA, USA, 250 mm × 4.6 mm, 5 *μ*m) was used. The sample injection volume was 20 *μ*l. The mobile phase was comprised of methanol and 40 mM ammonium dihydrogen phosphate (50 : 50, *v*/*v*). The total flow rate was 1 ml/min. The wavelength of detection was 280 nm. The temperature of the column was 35°C.

### 2.4. In Vivo Experiments

The mice were raised in specific pathogen-free environment at 22°C to 25°C, 50% humidity, and 12 h dark/light cycle. Food (AIN-93G) and water were being fed regularly.

#### 2.4.1. Intramyocardial Gene Delivery

Mice were intraperitoneally injected with 100 mg/kg ketamine and 8 mg/kg xylazine for anesthesia. The endotracheal intubation was performed, and then of the heart was exposed through the left anterior lateral incision of the fourth intercostal space. pAD/VDAC1 (2 × 10^11^ plaque-forming units/ml) was directly injected into the left ventricular free wall (4-5 sites, 10 *μ*l/site); the residual air was exhausted before closing the chest. Sham-operated mice underwent the same procedures except for the gene delivery [20].

#### 2.4.2. Preparation of Langendorff Isolated Heart Perfusion and A/R Injury Model

24 h post treatment, mice were anaesthetized by intraperitoneal injection of 100 mg/kg ketamine and 8 mg/kg xylazine. The heart was quickly taken out and kept in precooled Krebs-Henseleit (KH) buffer. Then, the heart was mounted on an improved Langendorff device and perfused with KH buffer saturated with 95% O_2_ and 5% CO_2_, at 37°C (pH 7.4) under 60-70 mmHg pressure. Carefully inserted a ball filled with water (6-10 mmHg) into the left ventricle. Hemodynamic parameters, including left ventricular developed pressure (LVDP, kPa), maximum positive and negative changes in LVDP (±*dp*/*dt* max, kPa/s), and coronary flow (CF, ml/min), were measured with PowerLab system (ADInstruments, Sydney, Australia) [20].

Firstly, the hearts were perfused by the above method for 30 min. Then, the normal KH buffer was replaced with modified KH buffer with glucose removed and saturated with 95% N_2_ and 5% CO_2_ at 37°C and pH 6.8, for 30 min to induce whole heart ischemia. The normal KH buffer was restored for another 30 min to induce A/R injury. The control hearts were only perfused by normal KH buffer [20].

#### 2.4.3. Experimental Design

Mice were randomly divided into 8 groups, namely, (1) control, (2) A/R, (3) vinegar+A/R, (4) TMP+A/R, (5) vinegar+A/R+pAD/VDAC1, (6) TMP+A/R+pAD/VDAC1, (7) vinegar+A/R+Atr, and (8) TMP+A/R+Atr. Mice in groups (3), (5), and (7) were given 0.1 ml/10 g vinegar (Brand vinegar C) by gavage every day for 6 weeks. Mice in groups (4), (6), and (8) were given 6 mg/kg TMP by gavage every day for 6 weeks. At the beginning of the last two weeks, mice in groups (5) and (6) were injected with pAD/VDAC1 according to the methods described above. Mice in groups (7) and (8) were intraperitoneally injected with 5 mg/kg Atr [23]. Mice in the control and A/R groups were given equal volume of normal saline by gavage for 6 weeks.

#### 2.4.4. Determination of Hemodynamic Parameters and Related Enzyme Activities

Hemodynamic parameters were recorded [20]. Creatine phosphokinase (CPK) and lactate dehydrogenase (LDH) activities were determined with a Bio-Rad 680 microplate reader (Hercules, CA, USA) according to the kit manufacturer's guidelines (Jiancheng, Nanjing, China).

#### 2.4.5. Measurement of Myocardial Infarction or Apoptosis by TTC/TUNEL Staining

After reperfusion, half of the hearts of the mice randomly were selected and cut into 1 mm cross sections. 2,3,5-Triphenyltetrazolium chloride (TTC, Sigma-Aldrich) staining was carried out as described previously [20]. Briefly, the sections incubated with 1% TTC in PBS (pH 7.4) for 30 min at 37°C and stored overnight at room temperature in 10% formaldehyde. Then, the sections were photographed with a digital camera and images were analyzed using planimetry by Image Jo software (National Institutes of Health, Bethesda, MD, USA). The risk area was calculated as the total ventricular area minus the cavity.

At the same time, the risk area of the left ventricular tissue of the remaining half of the mouse hearts was fixed and embedded, and cut into 5 *μ*m sections. The terminal deoxynucleotidyl transferase-mediated nick end labeling (TUNEL, Promega, Madison, WI, USA) staining was performed to evaluate myocardial apoptosis as described previously [20]. TUNEL-positive cells were counted [23].

#### 2.4.6. Caspase-3 Activity Measurement

Caspase-3 activity in the myocardium was measured by caspase-3 activity assay kit (R&D, Minneapolis, Minnesota, USA), according to the instruction of the manufacturer.

#### 2.4.7. Determination of Myocardial Antioxidant Potential and Oxidative Stress Level

For evaluating the antioxidant potential, the ferric reducing antioxidant power (FRAP) of myocardial homogenate pretreated by vinegar/TMP was determined as previously described (Cell Biolabs, Santiago, CA, USA) [23]. Superoxide dismutase (SOD), catalase (CAT), glutathione peroxidase (GSH-Px) activities, and malondialdehyde (MDA) levels were determined according to the instructions (Jiancheng).

#### 2.4.8. Western Blots Assay

Proteins from the myocardial samples and cardiomyocytes were extracted with a protein extraction kit (Applygen Technologies, Beijing, China). Then, the protein content was quantified using the bicinchoninic acid protein assay kit (Thermo). Protein expression was analyzed with western blotting as previously described [29]. From each sample, proteins (30 *μ*g) were separated on a 12% SDS-PAGE gel and transferred onto the polyvinylidene fluoride membranes. After transfer, the membranes were blocked and incubated overnight at 4°C with the following primary antibodies: VDAC1 (1 : 500), *cyt c* (1 : 500), and *β*-actin (1 : 2000). Secondary antibody by conjugated horseradish peroxidase (1 : 5000) was used, and *β*-actin was used as internal control.

### 2.5. In Vitro Experiments

#### 2.5.1. Primary Cardiomyocyte Culture

Cardiomyocytes from 0-3-day-old SD rats were isolated as previously described [18, 29]. Briefly, the hearts from neonatal rats were removed and placed in precooling D-Hanks' balanced salt solution. The ventricles were digested with 0.1% trypsin and then harvested repeatedly by centrifugation at 600 × *g* for 5 min. The cells were resuspended in plating medium (80% Dulbecco's minimal essential medium, DMEM), 20% fetal bovine serum, and 100 U/ml of penicillin and streptomycin, and plated in culture dishes that were incubated 37°C for 30 min to remove nonmyocytes. The suspended cells were plated on 60 mm gelatin-coated culture dishes at 1 × 10^6^ cells per dish and incubated at 37°C in a standard humidity incubator with 95% O_2_ and 5% CO_2_. After 18 h, cardiomyocytes were washed and plated in fresh medium and incubated for additional 3 days at 37°C in a standard humidity incubator with 95% O_2_ and 5% CO_2_ before the experiment.

#### 2.5.2. Adenovirus Transfection and A/R Damage

pAD/VCAD1 was transfected into cardiomyocytes cultured in fresh DMEM supplemented with 15% FBS. After 48 h, the transfection efficiency was about 85% [18]. The transfected cardiomyocytes were incubated at 37°C, 95% O_2_ and 5% CO_2_, for 2 h, and then, the subsequent experiments were carried out.

The culture plates with cardiomyocytes were placed in an air-tight anoxic chamber at 37°C, 95% N_2_ and 5% CO_2_, for 3 h, and then changed to 95% O_2_ and 5% CO_2_ for 2 h to induce A/R damage [18, 21].

#### 2.5.3. Experimental Design

Firstly, we need to eliminate the possible influence of acidity, confirm the concentration-effect relationship between vinegar/TMP pretreatment, and select the optimal concentration of vinegar/TMP. Cardiomyocytes were divided into the following groups: (1) control: cells were incubated in fresh DMEM for 50 h; (2) A/R: cells were incubated in fresh DMEM for 43 h, and then damaged by A/R with the above methods; (3) vinegar+A/R: cells were pretreated with various concentrations of vinegar (Brand vinegar C 1.25, 5, and 20 *μ*l/ml) for 43 h and transferred to fresh DMEM, followed by A/R damage; (4) TMP+A/R: cells were pretreated with various concentrations of TMP (5, 20, and 80 *μ*M) for 43 h and transferred to fresh DMEM, followed by A/R damage; and (5) acetic acid alone/acetic acid+A/R: cells were pretreated by 8% acetic acid 20 *μ*l/ml for 43 h and transferred to fresh DMEM, followed by A/R damage or normal DMEM. After relevant treatment, cell viability and LDH activity were measured.

Next, we explored whether VDAC1 expression could influence the effects of vinegar/TMP pretreatment against A/R damage. Cardiomyocytes were divided into the following groups: (1) control group; (2) A/R group; (3) vinegar+A/R group; (4) TMP+A/R group; (5) vinegar+A/R+pAD/VCAD1 group; (6) TMP+A/R+pAD/VCAD1 group; (7) vinegar+A/R+Atr group; and (8) TMP+A/R+Atr group. The cardiomyocytes of (3), (5), and (7) groups were pretreated with 5 *μ*l/ml vinegar (brand vinegar C) for 43 h and changed the fresh DMEM and suffered by A/R damage. The cardiomyocytes of (4), (6), and (8) groups were pretreated with 20 *μ*M TMP for 43 h and changed the medium and suffered by A/R damage. The cardiomyocytes of (5) and (6) groups were pretreated by pAD/VCAD1 for 2 h prior to the pretreatment with vinegar or TMP. The cardiomyocytes of (7) and (8) groups were pretreated by 50 *μ*M Atr for 2 h prior to A/R damage. After relevant treatment, cell viability, LDH and caspase-3 activities, apoptosis, VCAD1 expression, ROS generation, oxygen consumption rate (OCR), extracellular acidification rate (ECAR), mitochondrial membrane potential (MMP), mPTP openness, and release of *cyt c* to the cytoplasm were determined.

#### 2.5.4. Determination of Cell Viability, LDH and Caspase-3 Activities, Apoptosis, and Intracellular ROS Generation

After relevant treatment, LDH activity of supernatant was measured by LDH kits (Jiancheng). Cardiomyocytes were detected as follows: cell viability was tested by MTS kit (Promega), caspase-3 activity was detected by caspase-3 activity kit (R&D), apoptosis was measured with Annexin V-EGFP/PI apoptotic detection kit (BD Biosciences, San Diego, CA, USA), and intracellular ROS was assessed using a DCFH-DA probe (Invitrogen, Carlsbad, CA, USA), according to their manufacturer's instructions, respectively [15].

#### 2.5.5. Measurement of OCR and ECAR

Mitochondrial respiration and glycolysis may reflect bioenergetics and overall cell health. OCR and ECAR detected by XFp Extracellular Flux Analyzer (Seahorse Biosciences, North Billerica, MA, USA) could evaluate the above function in real time as previously described [18].

#### 2.5.6. Assessment of MMP and mPTP Opening

MMP loss was measured with the fluorescent probe JC-1 (Invitrogen, Carlsbad, CA, USA) and flow cytometry as previously described [18]. mPTP opening could be measured by the Ca^2+^-induced mitochondria swelling test as previously reported [23].

### 2.6. Statistical Analysis

Data were presented as the mean ± SEM. One-way or two-way analysis of variance with the post hoc Tukey-Kramer test was used to compare the groups. Statistical analysis was performed by Statistical Package for the Social Sciences (SPSS) software version 22.0 (IBM Corporation, Armonk, NY, USA). *P* < 0.05 was considered as statistically significant.

## 3. Results

### 3.1. TMP Concentration in Commercial Food Vinegar

We found that the elution peak of TMP could be detected at 5.0 min of the retention time (Figure 1(b)). TMP peaks of 6 vinegar samples were identified and quantified (Figure 1(c)). As shown in Table 1, brand vinegar A had the lowest TMP concentration at 221.73 *μ*g/ml. Brand vinegar F had the highest TMP concentration at 650.50 *μ*g/ml. No TMP was detected in brand vinegar D. The average concentration of TMP of the other 5 vinegars is 456.07 ± 90.85 *μ*g/ml. In addition, we found that TMP concentration in brand vinegars was proportional to its storage time (calculated from the production date, Table 1), which was consistent with the research results of Xu et al. [30].

### 3.2. Protective Effects of Vinegar/TMP Pretreatment on the Isolated Mouse Heart against A/R Damage

**Figure 2:**
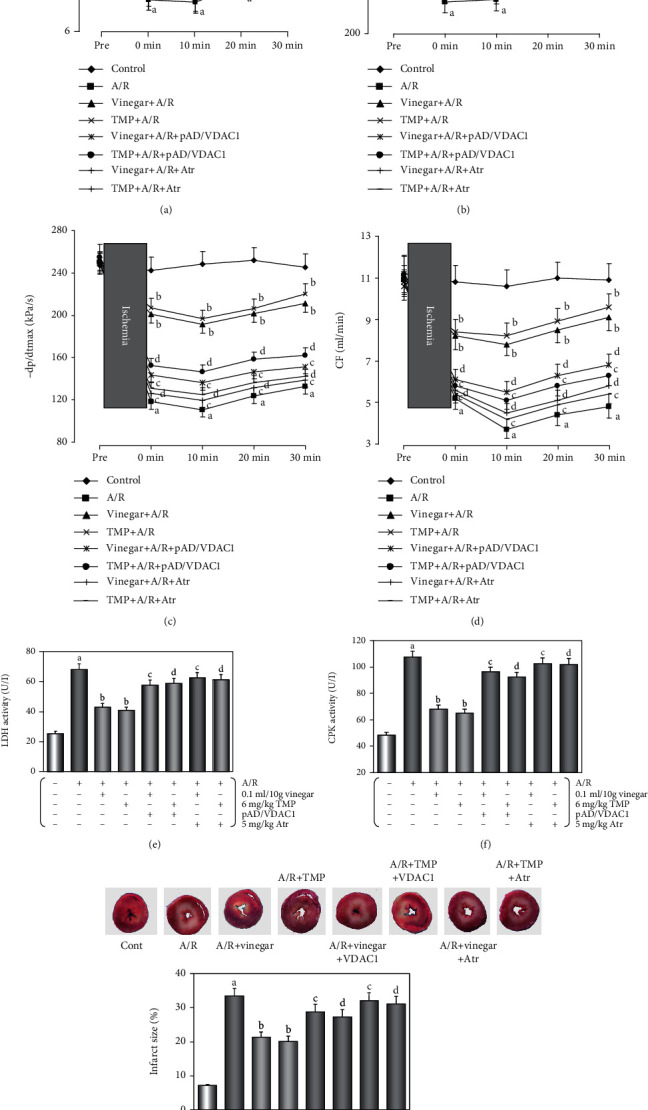
Effects of the hemodynamic parameters, LDH and CPK activities, and the myocardial infarct size by vinegar/TMP pretreatment on isolated mouse heart against A/R injury. (a) Histogram of LVDP. (b, c) Histogram of ±*dp*/*dt* max. (d) Histogram of CF. (e, f) Histogram of activities of LDH and CPK. Data were expressed as the mean ± SEM (*n* = 10). (g) Histogram of myocardial infarct size. Data were expressed as the mean ± SEM (*n* = 5). (a) *P* < 0.01 vs. the control group. (b) *P* < 0.01 vs. the A/R group. (c) *P* < 0.01 vs. the vinegar+A/R group. (d) *P* < 0.01 vs. the TMP+A/R group.

**Figure 3:**
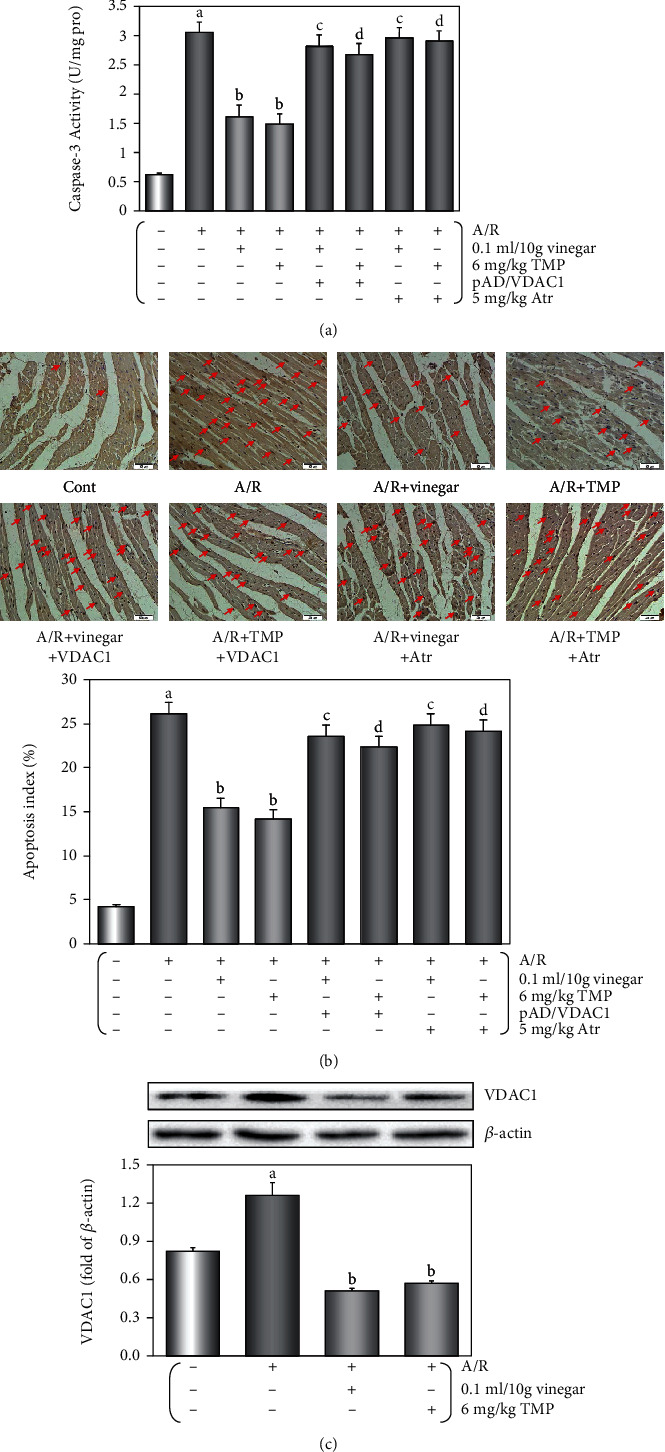
Effects of antiapoptotic and the expression of VCAD1 by vinegar/TMP pretreatment on isolated mouse heart against A/R injury. (a) Histogram of caspase-3 activity. (b) Histogram of apoptotic cells (TUNEL staining. Red arrows indicated TUNEL-positive cardiomyocytes). (c) The expression of VCAD1 in the myocardium. Data were expressed as the mean ± SEM (*n* = 5). (a) *P* < 0.01 vs. the control group. (b) *P* < 0.01 vs. the A/R group. (c) *P* < 0.01 vs. the vinegar+A/R group. (d) *P* < 0.01 vs. the TMP+A/R group.

Hemodynamic parameters such as LVDP, ±*dp*/*dt* max, and CF are important indicators of cardiac function [20]. After A/R damage, LVDP, ±*dp*/*dt* max, and CF significantly decreased; however, the cardiac function of mice was significantly recovered with vinegar/TMP pretreatment (Figures 2(a)–2(d), *P* < 0.01). Similarly, after A/R injury, the tissue damage-related enzyme LDH and CPK activities and the infarct size, which was the gold index for myocardial damage [23], all significantly increased, and still vinegar/TMP pretreatment could restore the changes (*P* < 0.01, Figures 2(e)–2(g)). Our results also showed a significant increase in caspase-3 activity and TUNEL-positive cells after A/R injury, which was attenuated by vinegar/TMP pretreatment (*P* < 0.01, Figures 3(a) and 3(b)). Interestingly, the protective effects of vinegar/TMP pretreatment were largely diminished by cotreatment of pAD/VDAC1 or Atr (*P* < 0.01, Figures 2 and 3).

It has been reported that the oxidative stress of the myocardium increases with A/R damage [19, 31]. Our results showed that SOD, CAT, and GSH-Px activities were inhibited, MDA level increased, and the FRAP results showed that myocardial antioxidant capacity decreased after A/R damage. Vinegar/TMP pretreatment could attenuate these adverse responses. However, these effects of vinegar/TMP pretreating were almost canceled by cotreatment of pAD/VDAC1 or Atr (*P* < 0.01, Table 2).

Western blot analysis (Figure 3(c)) indicated that A/R damage resulted in upregulation of VDAC1 (*P* < 0.01), and long-term oral vinegar/TMP intake significantly reduced it (*P* < 0.01).

The results showed that low-dose, long-term oral vinegar/TMP intake could induce protection to A/R injury to the isolated mouse heart and this protective effect is associated with the downregulation of VDAC1 expression in the myocardium and the opening of mPTP [20, 23].

### 3.3. Protective Effects of Vinegar/TMP Pretreatment on Cardiomyocyte against A/R Damage

**Figure 4:**
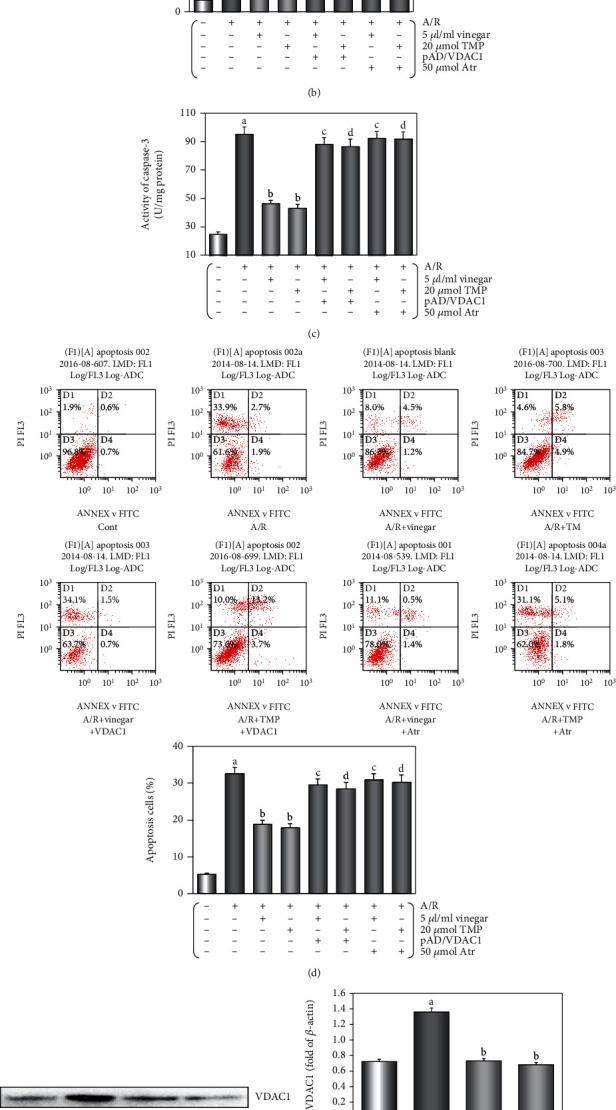
Protective effects of vinegar/TMP pretreatment for cardiomyocyte against A/R injury. (a) Histogram of cell viability. (b) Histogram of LDH activity. (c) Histogram of caspase-3 activity. (d) Flow cytometry dot plots and histogram of apoptotic cells. (e) Western blot of VCAD1 and *cyt c* expression in cardiomyocyte. (f) Histogram of VCAD1 expression. (g) Histogram of *cyt c* expression. Data were expressed as the mean ± SEM (*n* = 8). (a) *P* < 0.01 vs. the control group. (b) *P* < 0.01 vs. the A/R group; (c) *P* < 0.01 vs. the vinegar+A/R group. (d) *P* < 0.01 vs. the TMP+A/R group.

As shown in Supplemental Figures S1 and S2, cardiomyocytes subjected to A/R damage displayed decreased cell viability and increased LDH activity, and vinegar/TMP pretreating could reverse the changes in a concentration-dependent manner. In the acetic acid alone/acetic acid+A/R groups, the two indexes did not change, suggesting the observed protective effects from vinegar were independent of its acidity. The concentration of vinegar/TMP pretreating for the subsequent experiment was selected at 5 *μ*l vinegar/20 *μ*M TMP (Figures 4(a) and 4(b)).

Compared with the control group, cell viability/LDH activity did not alter in the groups with vinegar alone, TMP alone, pAD/VDAC1 alone, vinegar+pAD/VDAC1, TMP+pAD/VDAC1, vinegar+Atr, and TMP+Atr (*P* > 0.05), but decreased in the group with Atr alone (*P* < 0.01). The same was observed for the pAD/VDAC1+A/R group and Atr+A/R group compared with the A/R group (*P* < 0.01, Supplemental Figures S3 and S4), indicating that the upregulation of VDAC1 expression by pAD/VDAC1 and the opening of mPTP by Atr might aggravate cardiomyocyte damage [20, 23]. As shown in Supplemental Figure S5, in the group of vinegar/TMP alone, VDAC1 expression was significantly downregulated (*P* < 0.01). In the groups of pAD/VDAC1 alone and pAD/VDAC1+A/R, the expression of VDAC1 was upregulated to different degrees (*P* < 0.01, Supplemental Figure S6), indicating that adenovirus pAD/VDAC1 transfection was effective.

After A/R damage, caspase-3 activity and apoptosis rate were increased, whereas vinegar/TMP pretreatment significantly suppressed caspase-3 activity and apoptosis of cardiomyocyte (*P* < 0.01, Figures 4(c) and 4(d)). Similarly, the effects of vinegar/TMP pretreating were almost canceled with cotreatment of pAD/VDAC1 or Atr (*P* < 0.01, Figures 4(a)–4(d)).

Western blot analysis (Figures 4(e) and 4(f)) indicated that the changes of VDAC1 expression in cardiomyocytes were similar to those in the mouse myocardium.

### 3.4. Effects of Vinegar/TMP on Alleviating Mitochondrial Dysfunction in Cardiomyocyte

**Figure 5:**
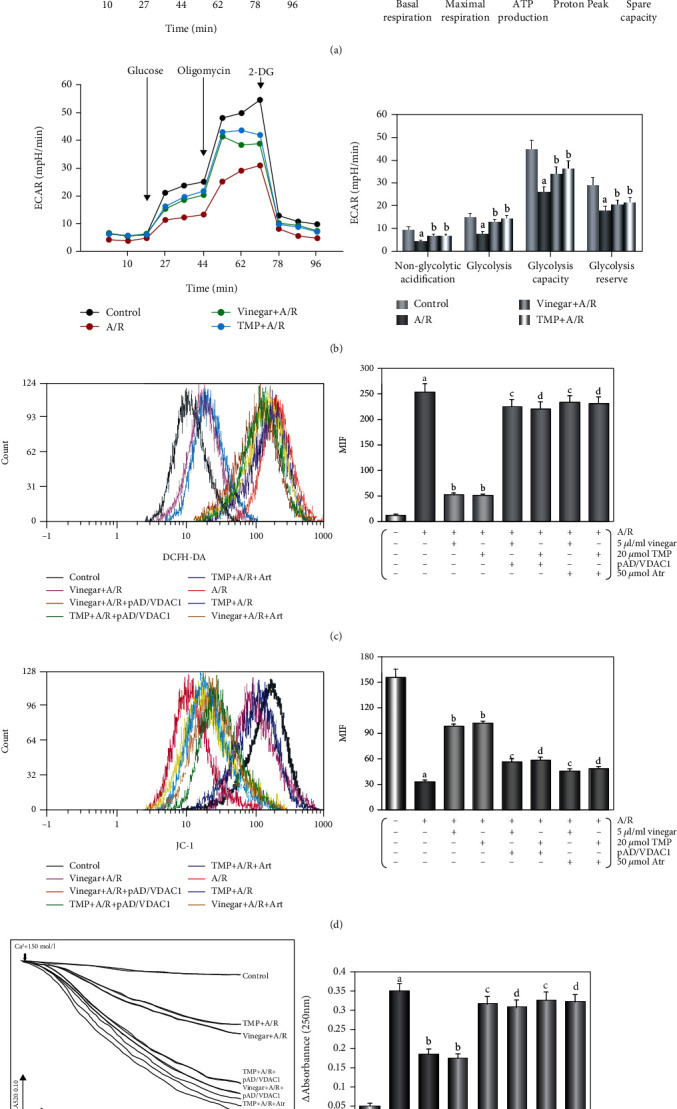
Effects of vinegar/TMP on alleviating mitochondrial dysfunction in cardiomyocyte against A/R injury. (a) Mitochondrial OCR curves and histogram of the important parameter. (b) Mitochondrial ECAR curves and histogram of the important parameter. Data were expressed as the mean ± SEM (*n* = 3). (c) Histogram of ROS generation. (d) Histogram of MMP levels. (e) Histogram of mPTP opening. Data were expressed as the mean ± SEM (*n* = 8). (a) *P* < 0.01 vs. the control group. (b) *P* < 0.01 vs. the A/R group. (c) *P* < 0.01 vs. the vinegar+A/R group. (d) *P* < 0.01 vs. the TMP+A/R group.

The OCR of cardiomyocyte by vinegar/TMP pretreatment was higher than those subjected to A/R. The results showed that the basal respiration, maximal respiration, ATP production, and spare respiratory capacity were significantly higher in cardiomyocytes undergone with vinegar/TMP pretreatment (*P* < 0.01, Figure 5(a)); however, the proton peak was slightly lower. Similarly, the ECAR of vinegar/TMP-pretreated cardiomyocyte was higher than A/R-pretreated cardiomyocyte, indicating significantly increased glycolysis and glycolytic capacity (*P* < 0.01, Figure 5(b)). But nonglycolytic acidification and glycolytic reserve increased slightly.

As shown, A/R damage caused a significant increase in ROS level. Vinegar/TMP pretreatment could significantly reduce ROS (*P* < 0.01, Figure 5(c)). In contrast, A/R damage caused a decline in MMP level, and vinegar/TMP pretreatment could reverse the change (*P* < 0.01, Figure 5(d)).

After A/R treatment, mitochondrial swelling was observed, indicating that mPTP was opened; vinegar/TMP pretreatment with mPTP opening showed a marked mild trend (*P* < 0.01, Figure 5(e)). In addition, A/R injury increased *cyt c* concentration in the cytoplasm (*P* < 0.01), and vinegar/TMP pretreatment significantly reduced it (*P* < 0.01, Figures 4(e) and 4(g)).

Similarly, these effects of vinegar/TMP pretreating were almost canceled with cotreatment of pAD/VCAD1 or Atr (*P* < 0.01, Figures 5(c)–5(e)).

## 4. Discussion

Vinegar is a worldwide popular food condiment and pickling material due to its taste and flavor [7]. It also has nutrition and health care functions [4, 6] and plays an important role in traditional Chinese medicine (TCM) food therapy [5] and Mediterranean diet [3]. Studies have shown that vinegar possesses antioxidant [6, 32], anti-inflammatory, and antiadiposity properties [3, 33], which may affect lipid profile, suppress adipocyte differentiation and fat accumulation, reduce body weight and plasma triglyceride, and prevent HFD-induced obesity and obesity-related cardiovascular complications [3, 6, 32–35]. Many healthy ingredients have been reported in vinegar, such as carbohydrates, organic acids, amino acids, peptides, and some functional factors, including TMP. TMP has pleasant tones of nutty and roasted flavors and is usually used to enhance food special flavor [2]. TMP is considered as one of the most aroma-active compounds [8, 36] and a functional ingredient [2, 37], determining vinegar quality [1, 28]. The formation mechanism of TMP in vinegar is controversial, but its concentration increases significantly with the storage time [30], which was reconfirmed in this study (Table 1, from 221.73 *μ*g/ml to 650.50 *μ*g/ml).

TMP is an alkaloid with multitarget and multimechanism. It is also extracted from *Ligusticum wallichii*'s rhizome. It has many biological functions, including inhibition of oxidative stress and inducing cytoprotection [9]. TMP is a promising candidate for managing cardiovascular and cerebrovascular diseases [10–12]. Our previous study also articulated that TMP has excellent protective function on a variety of the myocardium and blood vessel injuries [13–16]. In this study, we characterized the effect of vinegar/TMP pretreatment in alleviating A/R-induced myocardial damage by multiple functional, enzymatic, cellular, or molecular biological indicators (Figures 2–5, Table 2). It is worth noting that, firstly, by adopting a high concentration acetic acid as control [30], we ruled out the impact of vinegar on myocardial protection due to acidity. Secondly, similar levels of myocardial protection were observed between pretreatment of TMP and pretreatment of vinegar containing equivalent TMP concentration, implying TMP to be the most important myocardial protective factors in vinegar. Thirdly, A/R injury was induced only after complete clearance of the vinegar/TMP in both the *in vitro* (after media change) and *in vivo* (24 h after administration [9]) models. Thus, the observed myocardial protection was likely due to the change of cardiomyocytes' property during long-term NPC [17], rather than spontaneous response to the vinegar/TMP. Lastly, in this study, physiologically relevant concentration of vinegar was applied in priming to mimic the daily vinegar intake of people [4]. The results suggested that normal dietary consumption of vinegar (15 ml daily) meets the condition of NPC to induce myocardial protection [38].

IPC or PPC may trigger the production of endogenous myocardial protective substances, or change the related bioactive molecules to withstand serious I/R injury [17, 19]. However, the ethical concerns and technical difficulties are the limiting factors of these two methods. NPC is a convenient and feasible substitute, as it provides similar myocardial protection through dietary intake of natural and nontoxic nutrients [20]. In previous studies, we have demonstrated the use of several nutrients (including TMP, ferulic acid, resveratrol, curcumin, astragaloside IV, and apigenin) for NPC to effectively and safely protect the myocardium and blood vessels against various injuries [14, 20–24]. Therefore, it could be concluded that in this study, the effects of vinegar/TMP pretreatment belong to NPC myocardial protection. We further found that A/R injury could significantly upregulate VDAC1 expression, and with the protective effects induced by vinegar/TMP pretreatment, the upregulation of VDAC1 expression was significantly inhibited (Figures 3(c) and 4(e)); however, when combined with the pAD/VDAC1 to reupregulate VDAC1 expression, the protective effects of vinegar/TMP pretreatment were basically reversed (Figures 2–5). Therefore, it could be concluded that the target of vinegar/TMP pretreatment was VDAC1.

VDAC1 is involved in the formation of an important pore in the mitochondrial membrane, namely, mPTP, which is responsible for the transport of metabolites and signal transduction [25, 39]. VDAC1 is upregulated in A/R-injured cardiomyocytes. It forms complex with Bax, and triggers apoptosis through the release of apoptosis factor such as *cyt c* into the cytoplasm [40]. In the previous studies, we also obtained similar results: VDAC1 was upregulated in cardiomyocytes by A/R injury, which promoted mitochondrial-mediated apoptosis. Resveratrol, another important functional factor, could inhibit the upregulation and modification of VDAC1 and played a role in myocardial protection [20, 26, 27].

After A/R damage, ROS may break out with the increase of free radical and the decrease of scavenging ability [17–19, 31]. Our results found that after A/R injury, the myocardium showed the higher level of oxidative stress, weaker antioxidant potential, and more intracellular ROS generation. Due to TMP possessing stronger antioxidant capacity itself [9], vinegar/TMP pretreatment could partially reverse the above changes (Table 2, Figure 5(c)).

In recent years, it has been found that the mitochondria not only undertake and complete energy metabolism but also actively or passively participate in, or even determine cell function, survival state and death mode [41]. Therefore, to ensure the integrity of its structure and function is the basis of many life activities [23]. mPTP is a multiprotein complex formed between the inner and outer mitochondrial membranes. mPTP opening is regarded as a pivotal event in the irreversible reperfusion injury. Sustained mPTP opening will result in mitochondrial depolarisation, swelling, and rupture of the external membranes, and ultimately lead to mitochondrial dysfunction [42, 43]. As an important protein of mPTP, VDAC1 is located in the outer membrane of the mitochondria, which controls the entry and exit of mitochondrial metabolites and ions, as well as mPTP opening [25]. Therefore, inhibiting the function of VDAC1 can prevent mPTP opening, which is beneficial to the prevention of myocardial injury [20]. In the previous studies, we found that TMP could target the mitochondria, could prevent mPTP openness, and had an excellent protective effect on a variety of the myocardium or blood vessel injuries [13–15]. In the present study, with the downregulation of VDAC1 expression by vinegar/TMP pretreatment, the mitochondrial function of cardiomyocytes stimulated by A/R injury was significantly improved (Figure 5, including energy metabolism maintenance, acidosis correction, oxidative stress inhibition, and normal membrane function); however, the protection of vinegar/TMP pretreatment could be almost canceled by opening mPTP with Atr [23]. Therefore, the mitochondrion was the ultimate target organelle of vinegar/TMP pretreatment.

**Figure 6:**
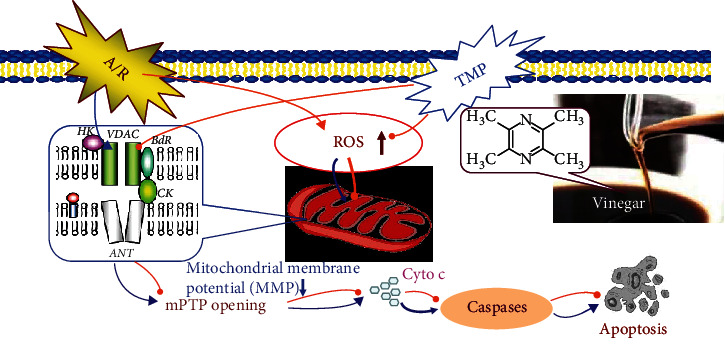
The mechanism of myocardial protection induced by vinegar/TMP. Vinegar contains TMP. TMP is one of the most important myocardial protective substances in vinegar. “Nutritional preconditioning” through low-dose long-term consumption of vinegar/TMP is shown to trigger myocardial protection, alleviating A/R-induced myocardial damage *in vivo* and *in vitro*. Vinegar/TMP pretreatment acts through downregulating VDAC1 expression, inhibiting oxidative stress, and preventing mitochondrial dysfunction. VDAC1 is their target, and the mitochondria are their target organelles.

In conclusion, the study reconfirmed the existence of TMP in vinegar and a varying concentration associated with storage time. “Nutritional preconditioning” through low-dose long-term consumption of vinegar/TMP was shown to trigger myocardial protection, alleviating A/R-induced myocardial damage *in vivo* and *in vitro* (Figure 6). We also demonstrated that TMP acts through downregulating VDAC1 expression, inhibiting mPTP opening, and preventing mitochondrial dysfunction.

## Figures and Tables

**Table 1:** Determination of TMP concentration in six different brands of vinegar (*n* = 3).

Sample no.	Peak area	Mean	Concentration (*μ*g/ml)	Storage time of mark (month)
Brand vinegar A	390.8	394.3	221.73	32
	394.9			32
	397.2			32
Brand vinegar B	451.0	448.4	261.22	40
	450.2			40
	444.1			40
Brand vinegar C	994.2	1006.1	512.59	62
	993.4			62
	1030.8			62
Brand vinegar D	No peak	—	—	
	No peak			
	No peak			
Brand vinegar E	1219.6	1214.7	634.33	70
	1217.3			70
	1207.1			70
Brand vinegar F	824.3	821.4	650.50	76
	813.3			76
	826.7			76

**Table 2:** Vinegar/TMP preconditioning preserves the myocardial homogenate ferric reducing antioxidant potentiality and the activities of antioxidant enzymes and reduced the levels of lipid peroxidation in the myocardium against A/R injury.

Groups	FRAP (mmol Fe^2+^/l)	SOD activity (U/g tissue)	GPx activity (U/g tissue)	CAT activity (U/g tissue)	MDA content (nmol/g tissue)
Control	5.11 ± 0.20	86.32 ± 4.22	19.52 ± 1.46	14.68 ± 1.25	29.06 ± 1.83
A/R	1.42 ± 0.04^a^	20.82 ± 1.61^a^	4.83 ± 0.51^a^	6.35 ± 0.52^a^	140.28 ± 7.52^a^
Vinegar+A/R	4.06 ± 0.23^b^	51.61 ± 2.62^b^	13.06 ± 1.35^b^	10.28 ± 0.82^b^	82.31 ± 5.06^b^
TMP+A/R	4.35 ± 0.22^b^	56.28 ± 2.81^b^	15.20 ± 1.38^b^	11.06 ± 0.76^b^	78.62 ± 4.91^b^
Vinegar+A/R+pAD/VDAC1	1.93 ± 0.06^c^	25.12 ± 1.85^c^	6.62 ± 0.60^c^	7.21 ± 0.54^c^	135.20 ± 7.08^c^
TMP+A/R+pAD/VDAC1	2.15 ± 0.05^d^	27.32 ± 1.66^d^	6.91 ± 0.58^d^	7.02 ± 0.53^d^	132.61 ± 7.63^d^
Vinegar+A/R+Atr	1.71 ± 0.05^c^	22.08 ± 1.58^c^	4.51 ± 0.61^c^	5.86 ± 0.55^c^	146.65 ± 8.02^c^
TMP+A/R+Atr	1.86 ± 0.06^d^	21.39 ± 1.62^d^	4.92 ± 0.57^d^	5.98 ± 0.58^d^	143.28 ± 7.31^d^

Data were presented as the mean ± SEM for five individual experiments. ^a^*P* < 0.01 vs. the control group. ^b^*P* < 0.01 vs. the A/R group. ^c^*P* < 0.01 vs. the vinegar+A/R group. ^d^*P* < 0.01 vs. the TMP+A/R group.

## Data Availability

The data used to support the findings of this study are included within the article.

## Abbreviations

A/R: Anoxia/reoxygenation
Atr: Atractyloside
CAT: Catalase
CPK: Creatine phosphokinase
cyt c: Cytochrome c
ECAR: Extracellular acidification rate
FRAP: Ferric reducing antioxidant power
GSH-Px: Glutathione peroxidase
HPLC: High-performance liquid chromatography
IPC: Ischemic preconditioning
I/R: Ischemia/reperfusion
K-H: Krebs-Henseleit
LDH: Lactate dehydrogenase
LVDP: Left ventricular developed pressure
MDA: Malondialdehyde
MMP: Mitochondrial membrane potential
mPTP: Mitochondrial permeability transition pore
NPC: Nutrition preconditioning
OCR: Oxygen consumption rate
PPC: Pharmacological preconditioning
ROS: Reactive oxygen species
SOD: Superoxide dismutase
TMP: Tetramethylpyrazine
TUNEL: Terminal deoxynucleotidyl transferase-mediated nick end labeling
VDAC: Voltage-dependent anion-selective channel.

## Supplementary Materials

Supplementary Materials Figure S1: vinegar/TMP pretreatment protects cardiomyocyte against A/R injury (on the cell viability). Vinegar/TMP pretreatment significantly increased the cell viability (*P* < 0.01) in a concentration-dependent manner. However, in the acetic acid alone/acetic acid+A/R group, the cell viability did not change (*P* > 0.05). Data were presented as the mean ± SEM for eight individual experiments. (a) *P* < 0.01 vs. the control group. (b) *P* < 0.01 vs. prior dosage. (c) *P* > 0.05 vs. the corresponding vinegar pretreatment. (d) *P* > 0.05 vs. the A/R group. (e) *P* > 0.05 vs. the control group. Figure S2: vinegar/TMP pretreatment protects cardiomyocyte against A/R injury (on the LDH activity). Vinegar/TMP pretreatment significantly increased the LDH activity (*P* < 0.01) in a concentration-dependent manner. However, in the acetic acid alone/acetic acid+A/R group, the LDH activity did not change (*P* > 0.05). Data were presented as the mean ± SEM for eight individual experiments. (a) *P* < 0.01 vs. the control group. (b) *P* < 0.01 vs. prior dosage. (c) *P* > 0.05 vs. the corresponding vinegar pretreatment. (d) *P* > 0.05 vs. the A/R group. (e) *P* > 0.05 vs. the control group. Figure S3: effects of vinegar/TMP alone, or upregulating VDAC1 expression, or opening mPTP on the cell viability of cardiomyocyte. Cell viability did not change by using vinegar alone, TMP alone, pAD/VDAC1 alone, vinegar+pAD/VDAC1, TMP+pAD/VDAC1, vinegar+Atr, and TMP+Atr when compared with the control group (*P* > 0.05). However, the cell viability with Atr alone was lower compared to that of the control group (*P* < 0.01), which was also the case for the pAD/VDAC1+A/R group and Atr+A/R group compared with the A/R group, indicating that treatment with pAD/VDAC1 upregulated VDAC1 expression, allowing Atr to open the mPTP, thereby aggravating cardiomyocyte injury. Data were presented as the mean ± SEM for eight individual experiments. (a, b) *P* < 0.01 vs. the control group. (c) *P* < 0.01 vs. the A/R group. Figure S4: effects of vinegar/TMP alone, or upregulating VDAC1 expression, or opening mPTP on the LDH activity of cardiomyocyte. LDH activity did not change by using vinegar alone, TMP alone, pAD/VDAC1 alone, vinegar+pAD/VDAC1, TMP+pAD/VDAC1, vinegar+Atr, and TMP+Atr when compared with the control group (*P* > 0.05). However, the LDH activity with Atr alone was higher compared to that of the control group (*P* < 0.01), which was also the case for the pAD/VDAC1+A/R group and Atr+A/R group compared with the A/R group, indicating that treatment with pAD/VDAC1 upregulated VDAC1 expression, allowing Atr to open the mPTP, thereby aggravating cardiomyocyte injury. Data were presented as the mean ± SEM for eight individual experiments. (a, b) *P* < 0.01 vs. the control group. (c) *P* < 0.01 vs. the A/R group. Figure S5: effects of vinegar/TMP alone on VDAC1 expression of cardiomyocyte. Vinegar/TMP alone significantly downregulated VDAC1 expression of the normal cardiomyocyte. Data were presented as the mean ± SEM for three individual experiments. (a) *P* < 0.01 vs. the control group. Figure S6: effects of pAD/VDAC1 alone treatment or pAD/VDAC1+A/R treatment on VDAC1 expression of cardiomyocyte. With pAD/VDAC1 alone treatment and pAD/VDAC1+A/R treatment, the expression of VCAD1 was upregulated in different degrees (*P* < 0.01), indicating that adenovirus pAD/VDAC1 could do good work. Data were presented as the mean ± SEM for three individual experiments. (a, b) *P* < 0.01 vs. the control group. (c) *P* < 0.01 vs. the A/R group.

## References

[1] Wu J., Zhao H., Du M., Song L., Xu X. (2019). Dispersive liquid-liquid microextraction for rapid and inexpensive determination of tetramethylpyrazine in vinegar. *Food Chemistry*.

[2] Xiao Z., Zhao L., Tian L., Wang L., Zhao J. (2018). GC-FID determination of tetramethylpyrazine and acetoin in vinegars and quantifying the dependence of tetramethylpyrazine on acetoin and ammonium. *Food Chemistry*.

[3] Bounihi A., Bitam A., Bouazza A., Yargui L., Koceir E. A. (2017). Fruit vinegars attenuate cardiac injury via anti-inflammatory and anti-adiposity actions in high-fat diet-induced obese rats. *Pharmaceutical Biology*.

[4] Chen J., Tian J., Ge H., Liu R., Xiao J. (2017). Effects of tetramethylpyrazine from Chinese black vinegar on antioxidant and hypolipidemia activities in HepG2 cells. *Food and Chemical Toxicology*.

[5] Zou P. (2016). Traditional Chinese medicine, food therapy, and hypertension control: a narrative review of Chinese literature. *The American Journal of Chinese Medicine*.

[6] Bouazza A., Bitam A., Amiali M., Bounihi A., Yargui L., Koceir E. A. (2016). Effect of fruit vinegars on liver damage and oxidative stress in high-fat-fed rats. *Pharmaceutical Biology*.

[7] Sakanaka S., Ishihara Y. (2008). Comparison of antioxidant properties of persimmon vinegar and some other commercial vinegars in radical-scavenging assays and on lipid oxidation in tuna homogenates. *Food Chemistry*.

[8] Liang J., Xie J., Hou L. (2016). Aroma constituents in Shanxi aged vinegar before and after aging. *Journal of Agricultural and Food Chemistry*.

[9] Donkor P., Chen Y., Ding L., Qiu F. (2016). Locally and traditionally used _Ligusticum_ species - A review of their phytochemistry, pharmacology and pharmacokinetics. *Journal of Ethnopharmacology*.

[10] Zhao Y., Liu Y., Chen K. (2016). Mechanisms and clinical application of tetramethylpyrazine (an interesting natural compound isolated from Ligusticum wallichii): current status and perspective. *Oxidative Medicine and Cellular Longevity*.

[11] Gao H. J., Liu P. F., Li P. W. (2015). Ligustrazine monomer against cerebral ischemia/reperfusion injury. *Neural Regeneration Research*.

[12] Hu J. Z., Huang J. H., Xiao Z. M., Li J. H., Li X. M., Lu H. B. (2013). Tetramethylpyrazine accelerates the function recovery of traumatic spinal cord in rat model by attenuating inflammation. *Journal of the Neurological Sciences*.

[13] Zhou Q., Chen S., Li H. (2020). Tetramethylpyrazine alleviates iron overload damage in vascular endothelium via upregulating DDAHII expression. *Toxicology In Vitro*.

[14] Yang B., Li H., Qiao Y. (2019). Tetramethylpyrazine attenuates the endotheliotoxicity and the mitochondrial dysfunction by doxorubicin via 14-3-3*γ*/Bcl-2. *Oxidative Medicine and Cellular Longevity*.

[15] Huang B., You J., Qiao Y. (2018). Tetramethylpyrazine attenuates lipopolysaccharide-induced cardiomyocyte injury via improving mitochondrial function mediated by 14-3-3*γ*. *European Journal of Pharmacology*.

[16] Chen H., He M., Huang Q., Zeng G., Liu D. (2007). Delayed protection of tetramethylpyrazine on neonatal rat cardiomyocytes subjected to anoxia-reoxygenation injury. *Basic & Clinical Pharmacology & Toxicology*.

[17] Hausenloy D. J., Yellon D. M. (2016). Ischaemic conditioning and reperfusion injury. *Nature Reviews Cardiology*.

[18] Qiao Y., Hu T., Yang B. (2020). Capsaicin alleviates the deteriorative mitochondrial function by upregulating 14-3-3*η* in anoxic or anoxic/reoxygenated cardiomyocytes. *Oxidative Medicine and Cellular Longevity*.

[19] Hausenloy D. J., Yellon D. M. (2011). The therapeutic potential of ischemic conditioning: an update. *Nature Reviews Cardiology*.

[20] Liao Z., Liu D., Tang L. (2015). Long-term oral resveratrol intake provides nutritional preconditioning against myocardial ischemia/reperfusion injury: involvement of VDAC1 downregulation. *Molecular Nutrition & Food Research*.

[21] Liao Z., He H., Zeng G. (2017). Delayed protection of ferulic acid in isolated hearts and cardiomyocytes: upregulation of heat-shock protein 70 via NO-ERK1/2 pathway. *Journal of Functional Foods*.

[22] He H., Luo Y., Qiao Y. (2018). Curcumin attenuates doxorubicin-induced cardiotoxicity via suppressing oxidative stress and preventing mitochondrial dysfunction mediated by 14-3-3*γ*. *Food & Function*.

[23] Luo Y., Wan Q., Xu M. (2019). Nutritional preconditioning induced by astragaloside IV on isolated hearts and cardiomyocytes against myocardial ischemia injury via improving Bcl-2-mediated mitochondrial function. *Chemico-Biological Interactions*.

[24] Huang H., Lai S., Luo Y. (2019). Nutritional preconditioning of apigenin alleviates myocardial anoxia/reoxygenation injury via mitochondrial pathway mediated by Notch1/Hes1. *Oxidative Medicine and Cellular Longevity*.

[25] Camara A. K. S., Zhou Y., Wen P. C., Tajkhorshid E., Kwok W. M. (2017). Mitochondrial VDAC1: a key gatekeeper as potential therapeutic target. *Frontiers in Physiology*.

[26] Tian M., Xie Y., Meng Y. (2019). Resveratrol protects cardiomyocytes against anoxia/reoxygenation via dephosphorylation of VDAC1 by Akt-GSK3 *β* pathway. *European Journal of Pharmacology*.

[27] Tong Z., Xie Y., He M. (2017). VDAC1 deacetylation is involved in the protective effects of resveratrol against mitochondria-mediated apoptosis in cardiomyocytes subjected to anoxia/reoxygenation injury. *Biomedicine & Pharmacotherapy*.

[28] Chen C., Chen Q., Guo Q. (2010). Simultaneous determination of acetoin and tetramethylpyrazine in traditional vinegars by HPLC method. *Food Chemistry*.

[29] He H., Zhou Y., Huang J. Y. (2017). Capsaicin protects cardiomyocytes against anoxia/reoxygenation injury via preventing mitochondrial dysfunction mediated by SIRT1. *Oxidative Medicine and Cellular Longevity*.

[30] Xu W., Xu Q., Chen J. (2011). Ligustrazine formation in Zhenjiang aromatic vinegar: changes during fermentation and storing process. *Journal of the Science of Food and Agriculture*.

[31] Cadenas S. (2018). ROS and redox signaling in myocardial ischemia-reperfusion injury and cardioprotection. *Free Radical Biology and Medicine*.

[32] Huang R., Huang Q., Wu G., Chen C., Li Z. (2017). Evaluation of the antioxidant property and effects in Caenorhabditis elegans of Xiangxi flavor vinegar, a Hunan local traditional vinegar. *Journal of Zhejiang University SCIENCE B*.

[33] Qui J., Ren C., Fan J., Li Z. (2010). Antioxidant activities of aged oat vinegar in vitro and in mouse serum and liver. *Journal of the Science of Food and Agriculture*.

[34] O’Keefe J. H., Gheewala N. M., O’Keefe J. O. (2008). Dietary strategies for improving post-prandial glucose, lipids, inflammation, and cardiovascular health. *Journal of the American College of Cardiology*.

[35] Ostman E., Granfeldt Y., Persson L., Björck I. (2005). Vinegar supplementation lowers glucose and insulin responses and increases satiety after a bread meal in healthy subjects. *European Journal of Clinical Nutrition*.

[36] Takuo K., Hiroshi Z., Kunio T., Takeshi Y., Hiroko N. (1971). Studies on flavor components of foodstuffs-part I. Distribution of tetramethylpyrazine in fermented foodstuffs. *Agricultural and Biological Chemistry*.

[37] Wang A., Song H., Ren C., Li Z. (2012). Key aroma compounds in Shanxi aged Tartary buckwheat vinegar and changes during its thermal processing. *Flavour and Fragrance Journal*.

[38] Abdukeyum G. G., Owen A. J., McLennan P. L. (2008). Dietary (n-3) long-chain polyunsaturated fatty acids inhibit ischemia and reperfusion arrhythmias and infarction in rat heart not enhanced by ischemic preconditioning. *The Journal of Nutrition*.

[39] Hseu Y. C., Thiyagarajan V., Ou T. T., Yang H. L. (2018). CoQ0-induced mitochondrial PTP opening triggers apoptosis via ROS-mediated VDAC1 upregulation in HL-60 leukemia cells and suppresses tumor growth in athymic nude mice/xenografted nude mice. *Archives of Toxicology*.

[40] Huckabee D. B., Jekabsons M. B. (2011). Identification of Bax-voltage-dependent anion channel 1 complexes in digitonin-solubilized cerebellar granule neurons. *Journal of Neurochemistry*.

[41] Bhatti J. S., Bhatti G. K., Reddy P. H. (2017). Mitochondrial dysfunction and oxidative stress in metabolic disorders -- A step towards mitochondria based therapeutic strategies. *Biochimica et Biophysica Acta (BBA)-Molecular Cell Research*.

[42] Boengler K., Lochnit G., Schulz R. (2018). Mitochondria “THE” target of myocardial conditioning. *American Journal of Physiology-Heart and Circulatory Physiology*.

[43] Hausenloy D. J., Ong S. B., Yellon D. M. (2009). The mitochondrial permeability transition pore as a target for preconditioning and postconditioning. *Basic Research in Cardiology*.

