# Supplementary material for: Vinegar/Tetramethylpyrazine Induces Nutritional Preconditioning Protecting the Myocardium Mediated by VDAC1
Source: Oxid Med Cell Longev. 2021 Apr 20;2021:6670088. doi: 10.1155/2021/6670088 (PMC8081599; doi:10.1155/2021/6670088)
Supplement: Supplementary Materials — Figure S1: vinegar/TMP pretreatment protects cardiomyocyte against A/R injury (on the cell viability). Vinegar/TMP pretreatment significantly increased the cell viability (P < 0.01) in a concentration-dependent manner. However, in the acetic acid alone/acetic acid+A/R group, the cell viability did not change (P > 0.05). Data were presented as the mean ± SEM for eight individual experiments. (a) P < 0.01 vs. the control group. (b) P < 0.01 vs. prior dosage. (c) P > 0.05 vs. the corresponding vinegar pretreatment. (d) P > 0.05 vs. the A/R group. (e) P > 0.05 vs. the control group. Figure S2: vinegar/TMP pretreatment protects cardiomyocyte against A/R injury (on the LDH activity). Vinegar/TMP pretreatment significantly increased the LDH activity (P < 0.01) in a concentration-dependent manner. However, in the acetic acid alone/acetic acid+A/R group, the LDH activity did not change (P > 0.05). Data were presented as the mean ± SEM for eight individual experiments. (a) P < 0.01 vs. the control group. (b) P < 0.01 vs. prior dosage. (c) P > 0.05 vs. the corresponding vinegar pretreatment. (d) P > 0.05 vs. the A/R group. (e) P > 0.05 vs. the control group. Figure S3: effects of vinegar/TMP alone, or upregulating VDAC1 expression, or opening mPTP on the cell viability of cardiomyocyte. Cell viability did not change by using vinegar alone, TMP alone, pAD/VDAC1 alone, vinegar+pAD/VDAC1, TMP+pAD/VDAC1, vinegar+Atr, and TMP+Atr when compared with the control group (P > 0.05). However, the cell viability with Atr alone was lower compared to that of the control group (P < 0.01), which was also the case for the pAD/VDAC1+A/R group and Atr+A/R group compared with the A/R group, indicating that treatment with pAD/VDAC1 upregulated VDAC1 expression, allowing Atr to open the mPTP, thereby aggravating cardiomyocyte injury. Data were presented as the mean ± SEM for eight individual experiments. (a, b) P < 0.01 vs. the control group. (c) P < 0.01 vs. the A/R group. Figure S4: effects of vi [file 6670088.f1.doc]

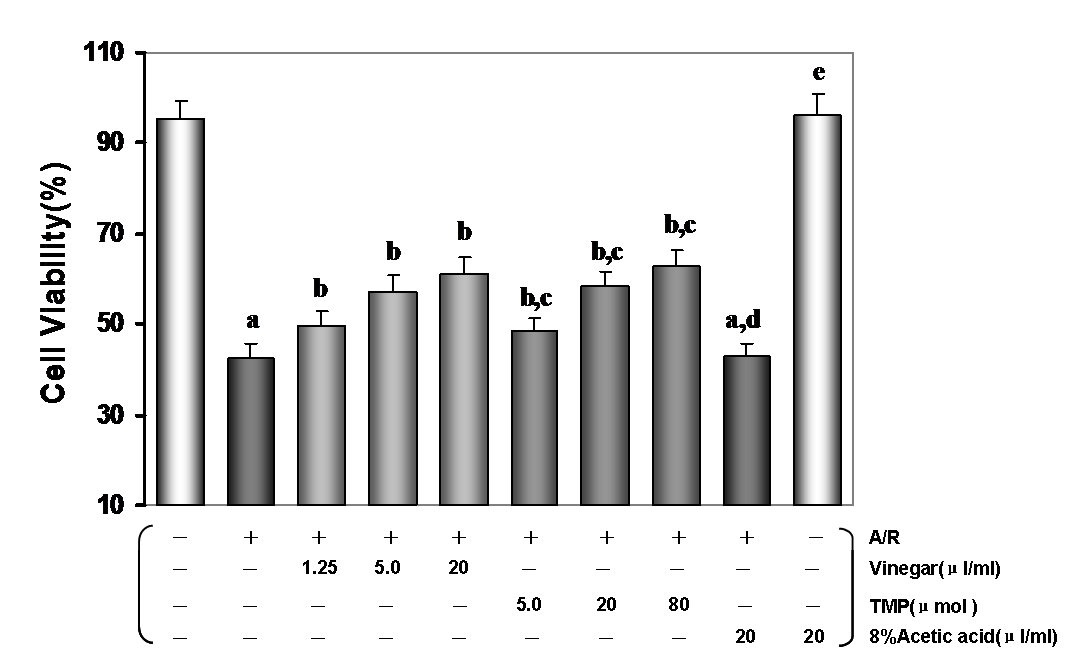


**Figure S1 Vinegar/TMP pretreatment protects cardiomyocyte against A/R injury (on the cell viability).** Vinegar/TMP pretreatment significantly increased the cell viability (*P* < 0.01) in a concentration-dependent manner. However, the Acetic acid alone/Acetic acid+A/R group, the cell viability did not change (*P* > 0.05). Data were presented as the mean ± SEM for eight individual experiments. a: *P* < 0.01, vs control group; b: *P* < 0.01, vs prior dosage; c: *P* > 0.05, vs the corresponding vinegar pretreatment; d: P > 0.05, vs A/R group; e: *P* > 0.05, vs control group.


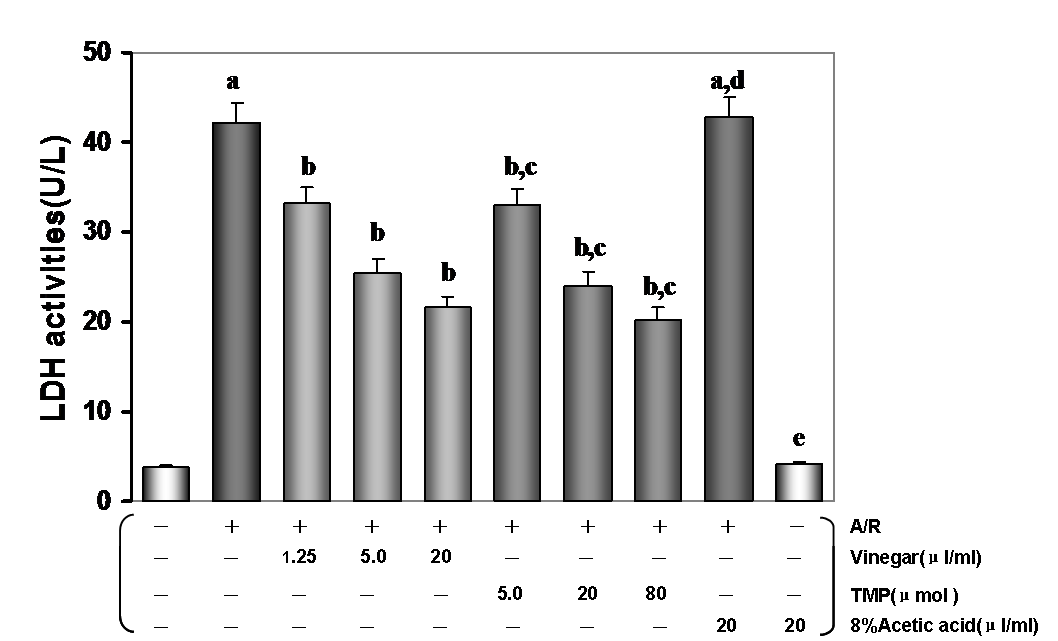


**Figure S2 Vinegar/TMP pretreatment protects cardiomyocyte against A/R injury (on the LDH activity).** Vinegar/TMP pretreatment significantly increased the LDH activity (*P* < 0.01) in a concentration-dependent manner. However, the Acetic acid alone/Acetic acid+A/R group, the LDH activity did not change (*P* > 0.05). Data were presented as the mean ± SEM for eight individual experiments. a: *P* < 0.01, *vs* control group; b: *P* < 0.01, *vs* prior dosage; c: *P* > 0.05, *vs* the corresponding vinegar pretreatment; d: *P* > 0.05, *vs* A/R group; e: *P* > 0.05, *vs* control group.


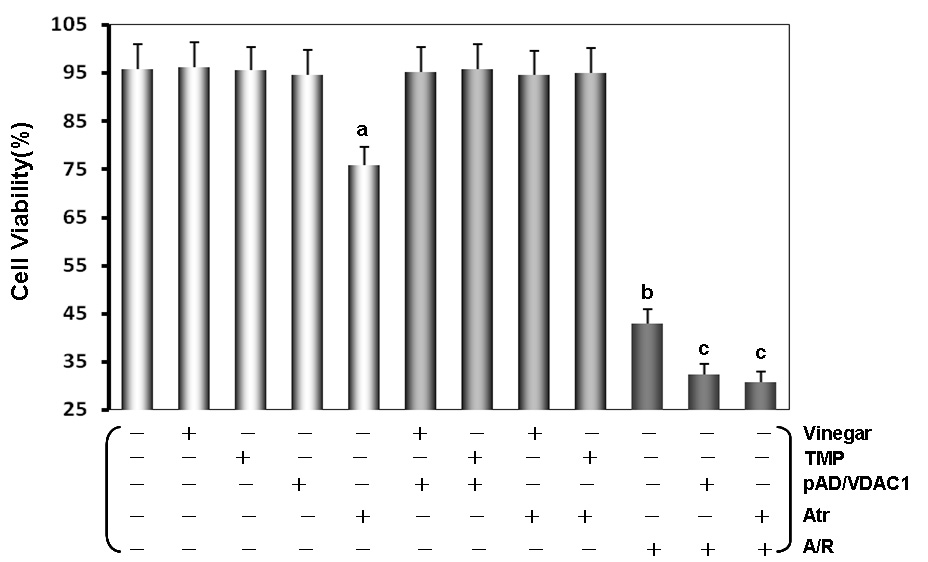


**Figure S3 Effects of vinegar/TMP alone, or upregulating VDAC1 expression, or opening mPTP on the cell viability of cardiomyocyte.** Cell viability did not change by using vinegar alone, TMP alone, pAD/VDAC1 alone, vinegar+pAD/VDAC1, TMP +pAD/VDAC1, vinegar+Atr, and TMP+Atr when compared with the control group (*P* > 0.05). However, the cell viability with Atr alone were lower compared to that of the control group (*P* < 0.01), which was also the case for the pAD/VDAC1+A/R group and Atr+A/R group compared with the A/R group, indicating that treatment with pAD/VDAC1 upregulated VDAC1 expression, allowing Atr to open the mPTP, thereby aggravating cardiomyocyte injury. Data were presented as the mean ± SEM for eight individual experiments. a, b: *P*<0.01, *vs* control group; c: *P*<0.01 *vs* A/R group.


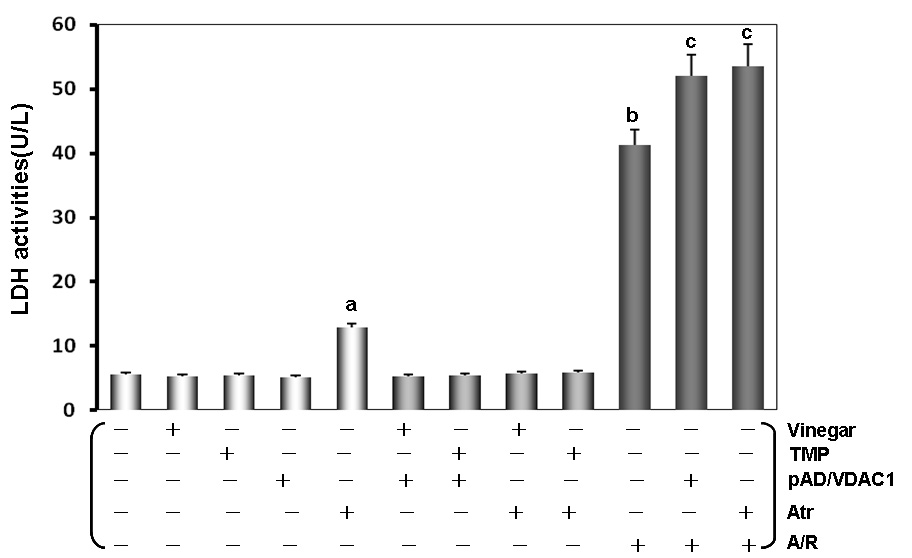


**Figure S4 Effects of vinegar/TMP alone, or upregulating VDAC1 expression, or opening mPTP on the LDH activity of cardiomyocyte.** LDH activity did not change by using vinegar alone, TMP alone, pAD/VDAC1 alone, vinegar+pAD/VDAC1, TMP +pAD/VDAC1, vinegar+Atr, and TMP+Atr when compared with the control group (*P* > 0.05). However, the LDH activity with Atr alone were higher compared to that of the control group (*P* < 0.01), which was also the case for the pAD/VDAC1+A/R group and Atr+A/R group compared with the A/R group, indicating that treatment with pAD/VDAC1 upregulated VDAC1 expression, allowing Atr to open the mPTP, thereby aggravating cardiomyocyte injury. Data were presented as the mean ± SEM for eight individual experiments. a, b: *P*<0.01, *vs* control group; c: *P*<0.01 *vs* A/R group.


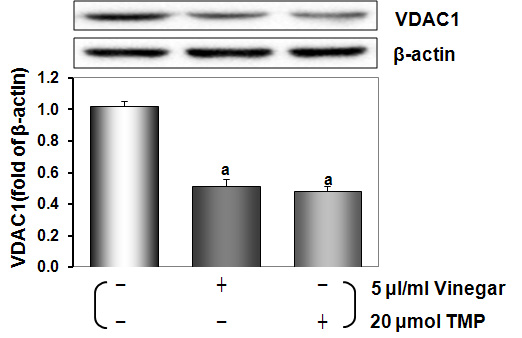


**Figure S5 Effects of vinegar/TMP alone on VDAC1 expression of cardiomyocyte.** Vinegar/TMP alone significantly downregulated VDAC1 expression of the normal cardiomyocyte. Data were presented as the mean ± SEM for three individual experiments. a, *P*<0.01, *vs* control group.


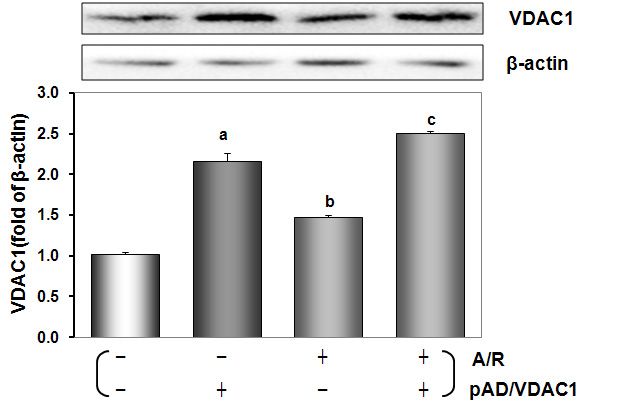


**Figure S6 Effects of pAD/VDAC1 alone treatment or pAD/VDAC1+A/R treatment on VDAC1 expression of cardiomyocyte.** With pAD/VDAC1 alone treatment and pAD/VDAC1+A/R treatment, the expression of VCAD1 was upregulated in different degrees (*P*<0.01), indicating that adenovirus pAD/VDAC1 could good work. Data were presented as the mean ± SEM for three individual experiments. a, b: *P*<0.01, *vs* control group; c: *P*<0.01 *vs* A/R group.
